# Predatory efficacy of five locally available copepods on *Aedes* larvae under laboratory settings: An approach towards bio-control of dengue in Sri Lanka

**DOI:** 10.1371/journal.pone.0216140

**Published:** 2019-05-28

**Authors:** Lahiru Udayanga, Tharaka Ranathunge, M. C. M. Iqbal, W. Abeyewickreme, Menaka Hapugoda

**Affiliations:** 1 Department of Biosystems Engineering, Faculty of Agriculture & Plantation Management, Wayamba University of Sri Lanka, Sri Lanka; 2 Molecular Medicine Unit, Faculty of Medicine, University of Kelaniya, Sri Lanka; 3 National Institute of Fundamental Studies, Kandy, Sri Lanka; 4 Department of Paraclinical Science, Faculty of Medicine, Sir John Kotelawala Defense University, Ratmalana, Sri Lanka; National Taiwan Ocean University, TAIWAN

## Abstract

Many countries are in search of more effective and sustainable methods for controlling dengue vectors, due to undeniable inefficiencies in chemical and mechanical vector control methods. Bio-control of vectors by copepods is an ideal method of using interactions in the natural ecosystem for vector management, with minimum consequences on the environment. Current study determined the predatory efficacy of five locally abundant copepod species on, *Aedes* larvae under laboratory conditions. Copepods were collected from the pre-identified locations within the districts of Gampaha and Kandy, and identified morphologically. Individual species of copepods were maintained as separate colonies with *Paramecium* culture and wheat grain as supplementary food. Five adult copepods of each species was introduced into separate containers with 200 larvae (1^st^ instar) of *Aedes aegypti*. Number of larvae survived in containers were enumerated at 3 hour intervals within a duration of 24 hours. Each experiment was repeated five times. The same procedure was followed for *Ae*. *albopictus*. Significance in the variations among predation rates was evaluated with General Linear Modelling (GLM) followed by Tukey’s pair-wise comparison in SPSS (version 23). Significant variations in predation rates of studied copepod species were reported (p<0.05), whereby *M*. *leuckarti* indicated the highest followed by *M*. *scrassus*, while *C*. *languides* indicated the lowest predatory efficacy. The effect of different *Aedes* larval species on the predation rates of copepods remained significant (p<0.05), even though the effect on predatory efficiency was not significant. Based on the findings, both *M*. *leuckarti* and *M*. *scrassus*, with the highest predatory efficiencies, could be recommended as potential candidates for biological controlling of *Aedes* vectors in Sri Lanka.

## Background

*Aedes aegypti* and *Ae*. *albopictus* mosquitoes, responsible for the transmission of dengue, are two of the most efficient transmitting agents of vector borne diseases [1]. At present, nearly 2.5 billion people living in more than 128 countries, are at risk from the incidence of dengue, which has accounted for approximately 390 million infections every year [2]. As declared by the World Health Organization, the Western Pacific and South-East Asia Regions remain as a hot spot for dengue, accounting for approximately 75% of the recent global disease burden of dengue [3].

The Health sector of Sri Lanka has been challenged by dengue, since mid-1960s, and with time, dengue has developed into a regular epidemic, becoming the worst threat to the health sector of the island [4]. In 2017, Sri Lanka witnessed the most severe outbreak of dengue by, having 186,101 patients with over 440 deaths [5]. Therefore, similar to many developing countries, Sri Lanka is also engaged in a fight against dengue to ensure the healthy status of the population.

The presence of four serotypes of the dengue virus, has complicated the development of a promising vaccine for dengue [6]. Therefore, controlling the mosquito vectors remains as the only effective approach for management of dengue outbreaks for many countries [7]. A variety of strategies ranging from chemical based controlling methods to integrated approaches and community participation are being considered for the suppression of *Aedes* vectors below the threshold levels of causing dengue epidemics [8]. However, the development of insecticide resistance among vectors [9], retention of insecticide residues in the natural environment [10–11], ill effects on humans and other biota [12] and unbalancing the functionality of ecosystems have influenced the consideration of the feasibility of other novel approaches for vector control [7]. On the other hand, mechanical methods used for source reduction of vectors, are often time and labour consuming and requires continuous human involvement, restricting the practical efficacy of such methods [13].

Therefore, a continuous search for more effective, ecofriendly and innovative methods could be seen among the Vector Controlling Entities (VCE) to restrict the density of adult and immature stages of *Aedes* mosquitoes [9]. Meanwhile, few novel strategies such as Sterile Insect Technique (SIT) and Incompatible Insect Technique (IIT), application of chitosan-synthesized silver nanoparticles (Ch–AgNP), green-fabricated nanoparticles as toxic agents against mosquito young instars, and as adult oviposition deterrents, have provided highly efficient approaches for controlling different *Aedes* species [14–16]. Regardless of the encouraging results provided by above novel strategies, practical implementation of such methods, especially in developing countries like Sri Lanka, is challenging due to restrictions in financial resources, expertise and the motivation of VCE & other government stakeholders. Hence, biological control, where locally available natural predators are used to target the immature or adult stages of vector mosquitoes, is an ideal cost-effective, environmentally friendly and effective strategy for controlling of vectors [13,17–18].

An animal that naturally preys on an inferior organism (prey) for food, is known as a predator. Predator–prey relationships remain as sophisticated interactions in all the ecosystems, since both predator and prey evolve together, developing physiological, morphological, behavioral, population and community interactions, to predate and avoid predation, respectively [19]. In the perspectives of the predator, prey is a part of its own environment, which should be consumed for survival. Hence, predators often develop a variety of adaptations such as, speed, stealth, camouflaging, sensory organs to detect prey through smell, sight, or hearing, immunity to the prey's poison, poison (to kill the prey) and appropriate organs for digestion of prey [20–21]. On the other hand, prey also co-evolve with the predator by having speed, accurate sensory organs, thorns and poison (to scape) to avoid being eaten. Both over predation of the prey or under predation, could results dramatic imbalances in the ecosystem, that might reshape and even collapse the considering ecosystem, due to other inter and intra species interactions. Use of a natural predator for biological controlling of *Aedes*, would provide a cost-effective yet efficient mechanism for controlling of *Aedes* vectors with minimal impacts on the environment [16]. However, selection of a natural predator should be done cautiously, while respecting the prey-predator interactions, evolutional, environmental and behavioral characteristics to predict possible scenarios [22–23].

A wide range of natural predators, ranging from microscopic to macroscopic, (dytiscid beetles, crustaceans, notonectids, belostomatids, Odonata, larvivorous fish and amphibians) have been used as biological control agents against different mosquito vectors in many parts of the world [24]. Being, micro-crustaceans, Cyclopoid copepods have often been used as effective biological control agents against different species of mosquitoes, such as *Anopheles* [25], *Aedes* [26–27] and *Culex* [26].

Since the first ever documentation of the larvivorous potential of copepods on *Aedes* by Riviere and Thirel [26], many countries have focused on biological control of dengue vectors by using copepods. Cyclopoid copepods such as *Macrocyclops albidus* [28], *Mesocyclops aspericornis* [26] *Mesocyclops australiensis* [29], *Mesocyclops darwini*, *Mesocyclops leuckuarti* [27, 30] and *Mesocyclops longisetus* [28] have shown their potential in suppressing the *Aedes* vector populations. Ability of inhabiting an array of diverse habitats, less economic requirements, high reproductive rates and resistance to some insecticides, high predacious nature on *Aedes* vectors have made copepods to play a key role in dengue epidemic management within many countries [28, 31–32]. Therefore, use of copepods, remains as a vital step in integrated vector management programmes in the recent years [31]. In a recent study, a combined application of *Gracilaria firma* synthesized silver nanoparticles and *M*. *formosanus* copepods, have resulted promising results against Aedes *aegypti*, providing an ecofriendly and sustainable approach for vector control [16].

With the natural location and environmental conditions of Sri Lanka, a high diversity of copepods inhabiting a wide array of freshwater lakes, reservoirs, streams, and ponds are found naturally. Even though, the efficacy of using copepods as biological control agents of vector mosquitoes has been advocated by numerous studies conducted throughout the world, potential of copepods for controlling *Aedes* larvae has not been sufficiently evaluated in Sri Lanka. Therefore, the current laboratory evaluation was conducted to evaluate the effectiveness of locally abundant copepods as biological control agents of *Aedes* vectors as an essential preliminary step towards more sustainable, cost-effective, efficient and ecofriendly management of dengue epidemics within the country.

## Methods

### Establishment of *Aedes* colonies

Adult mosquito surveillance activities were conducted within the Ragama Medical Officer of Health (MOH) area, Gampaha (7°12'7.56"N, 80°48'19.37"E) and all the collected mosquitoes were transported to the Molecular Medicine Unit, Faculty of Medicine, University of Kelaniya, Sri Lanka. Through morphological identification based on the keys elaborated by Rueda [32], two blood fed females of *Ae*. *aegypti* and *Ae*. *albopictus* were identified and reared in separate colonies, until oviposition. Eggs laid by above separated blood-engorged females were used to establish separate colonies of *Ae*. *aegypti* and *Ae*. *albopictus*. The established colonies were maintained in 24 x 24 x 24 cm cages, separately under standard conditions (at 27 ± 2° C and 75 ± 5% humidity) with a 12:12 (light:dark) cycle. The eggs laid by them were transferred from oviposition cups into hatching trays and allowed to be hatched, separately. First instar larvae [L_1_] of *Ae*. *aegypti* and *Ae*. *albopictus* were used for the predation trails [33].

### Establishment of copepod colonies

**Fig 1:**
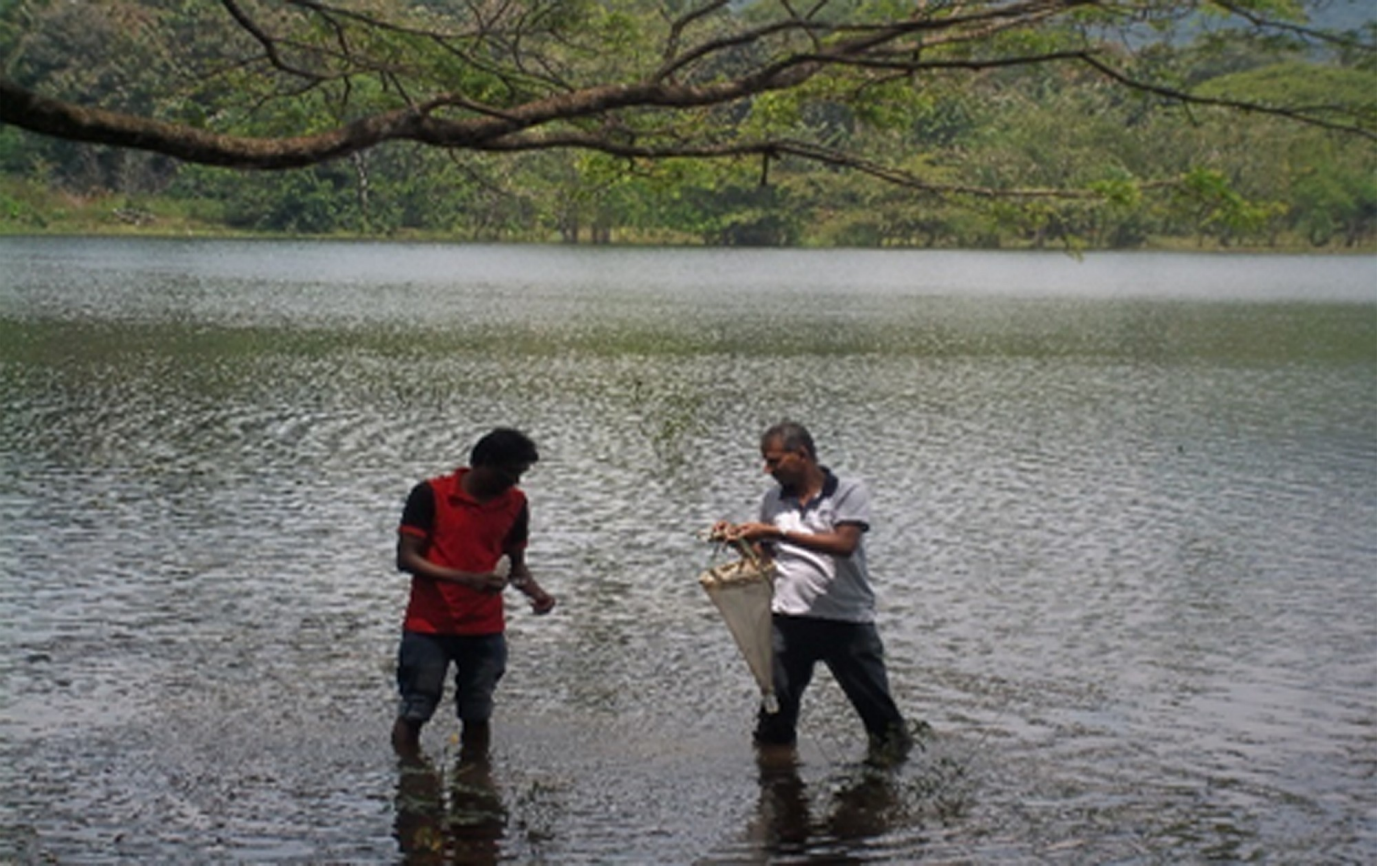
Collection of copepods from water bodies.

Copepods were collected from ponds, ditches and other standing water sources located within the districts of Gampaha (7°12'7.56"N, 80°48'19.37"E) and Kandy (7° 5'41.72"N, 79°59'59.64"E) by using a long-handled net (with 20 x 20 cm aperture, 100 μm mesh) at all depths (Fig 1). Since, the collections were made at public places and as the copepods were not a conservation priority in the country, a special permission was not required for field collection of copepods. Collected samples were poured through a sieve (with 2 mm mesh) to remove any debris and potential copepod predators and transported to the Molecular Medicine Unit within labelled glass containers. Collected copepods were identified to the species level under a light microscope (Olympus Optical Co. Ltd., Tokyo) with an objective (X l0) using standard keys [34–36]. One gravid female from each identified species was used to establish monocultures of separate copepod species in plastic containers with pond water (1 L). A mixture of *Paramecium* culture and wheat grain was supplied as food, while the cultures were maintained under a 12:12 (sunlight: dark) cycle at standard conditions (at 27 ± 2°C and 75 ± 5% humidity).

### Predation experiments

Five conspecific adult copepods were introduced into different larval rearing white coloured trays (25 × 25 × 7 cm) filled with deionized water (500 ml) and 200 *Ae*. *aegypti* larvae (1^st^ instar). The larvae remaining in each container and dead larvae were observed at 3 h intervals until 24 h at 27 ^o^C, under constant artificial lighting conditions. Finally the numbers of dead larvae were counted using a magnifying glass (X 10) every 3 h by three well trained entomological assistants and recordings of all the three operators were averaged to calculate mortality rate of *Aedes* larvae. In case where, more than 75% of *Aedes* larvae was predated, new batches of 1^st^ instar larvae were introduced to maintain the 200 of prey density for another interval. The whole experiment was repeated five times for each copepod species to maintain the accuracy of the findings. The same procedure was followed for *Ae*. *albopictus*.

In order to minimize the impact of external factors on the predation behavior of copepods, the larval trays were maintained in separate partitioned chambers (30 × 30 × 30 cm) made up with while coloured art boards (approximately 1 mm), and diffuse lighting systems were used for artificial lighting during the 12 h lighting period. Further, the entomological assistants that enumerated the larvae were wearing white coloured lab coats, and spent a minimum time (< 5 minutes) to count the surviving larvae (at 3 hour intervals), while ensuring minimum disturbance to larval trays. During the entire study period, human movements were limited to enumeration and reintroduction of larvae (where, necessary).

### Data interpretation and statistical analysis

The predation rates of different copepod species were calculated as the deducted product of remaining mosquito larvae from the initial/earlier surviving larvae for *Ae*. *aegypti* and *Ae*. *albopictus*, separately. Predatory efficiencies of different copepods were calculated by following the formula (Eq 1) introduced by Chitra *et al*. [37].

$$Predatory\;Efficiency=\frac{\left[Number\;of\;prey\;consumed/Number\;of\;predator\;introduced\right]}{Total\;number\;of\;prey\;introduced}X\;100$$ 1

General Linear Model (GLM) followed by Tukey’s pair-wise comparison in SPSS (version 23) was used for statistical comparison of the predation rates shown by different species of copepods. Predatory efficiencies of copepods were subjected to square-root transformation. A cluster analysis (with respect to Bray Curtis similarity) followed by Analysis of Similarities (ANOSIM) and Multi-Dimensional Scaling (MDS) was used for the visual representation and comparison of statistical significance in the predation patterns of the studied copepods in terms of overall predation on *Ae*. *aegypti* and *Ae*. *albopictus*. The Plymouth Routines in Multivariate Ecological Research version 6 (PRIMER 6) was used to perform the statistical comparisons.

## Results

### The predation rates of copepods on *Aedes* larvae

Five species of copepods, namely *Cyclops languides*, *C*. *varicans*, *C*. *vernalis*, *Mesocyclop leuckarti* and *Mesocyclop scrassus* were identified from the field collections. Among the studied candidates, *M*. *leuckarti* had the highest predation of *Ae*. *aegypti* (Mean ± Standard Error; 34.9±1.80) and *Ae*. *albopictus* (33.5±1.06) within 24 hours, followed by *Mesocyclop scrassus*. *C*. *languides* showed the lowest predation rate within 24 hours with a larvicidal potential of 10.6±1.60 and 8.4±1.10 for *Ae*. *aegypti* and *Ae*. *albopictus*, respectively (Table 1). As suggested by the results of the General Linear Model, the larvicidal potentials of the copepod species varied significantly (p<0.05 at 95% level of confidence).

**Table 1:** Mean number of Aedes larvae consumed by different copepod species in 24 hours.

Copepod Species	Average number of 1^st^ instar larvae consumed by a copepod within 24 hours	
	*Ae*. *aegypti*	*Ae*. *albopictus*
*Mesocyclop leuckarti*	34.9±2.2 ^a^ (32.7–37.1)	33.5±2.63 ^a^ (30.87–36.13)
*Mesocyclop scrassus*	31.0±2.7 ^b^ (28.3–33.7)	28.4±2.93 ^b^ (25.47–31.33)
*Cyclops vernalis*	17.1±1.4 ^c^ (15.7–18.5)	19.6±2.1 ^c^ (17.5–21.7)
*Cyclops varicans*	15.2±1.0 ^d^ (14.2–16.2)	12.8±1.2 ^d^ (11.6–14.0)
*Cyclops languides*	10.6±1.2 ^e^ (9.4–11.8)	8.4±1.5 ^e^ (6.9–9.9.)

**Note:** Values are Mean ± SE, range in parenthesis. Different superscript letters (from a to e) in a column show significant differences (p< 0.05) as suggested by General Linear Modelling followed by the Tukey’s pair wise comparison at 95% level of significance.

Results of the Tukey’s pair wise comparison (post-hoc analysis) denoted that the predation rates of all the copepods varied significantly from each other (p<0.05 at 95% level of confidence) as indicated in Table 1. Further, the two species of *Aedes* vectors, had a significant effect on the predation rates of different copepods (p>0.05 at 95% level of confidence). The predation rate of *Ae*. *aegypti* was significantly high, suggesting that *Ae*. *aegypti* is more preferred than *Ae*. *albopictus* as a dietary item by all the studied copepods

### Temporal variation of the larvicidal predation rates of copepods

**Fig 2:**
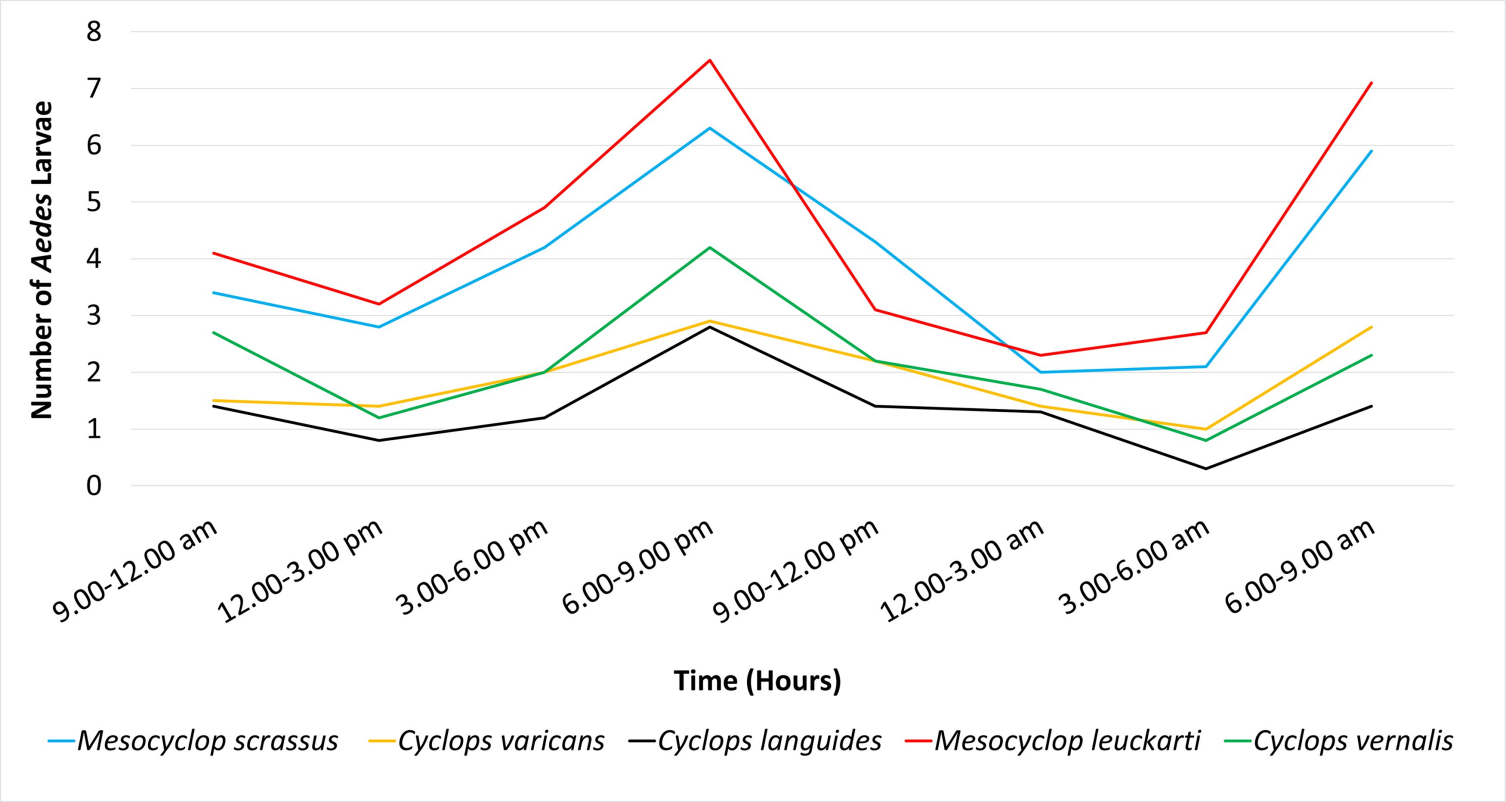
Temporal variation of the predation rates of studied copepods on *Ae*. *aegypti* larvae.

**Fig 3:**
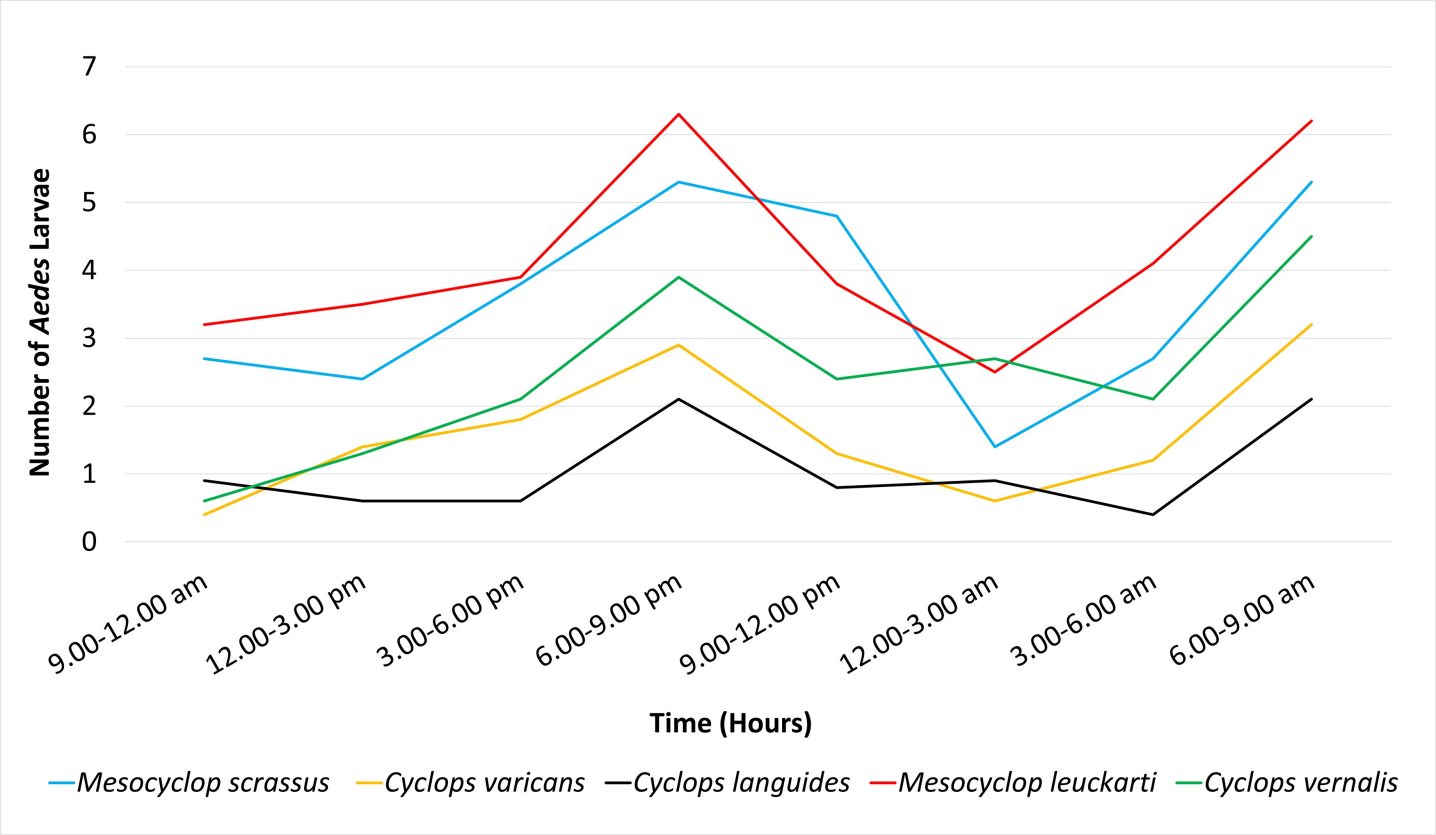
Temporal variation of the predation rates of studied copepods on *Ae*. *albopictus* larvae.

As, denoted by the Figs 2 and 3, two notable peaks of larval predation (on both *Ae*. *aegypti* and *Ae*. *albopictus*) was observed at dawn (6.00 a.m. to 9.00 a.m.) and dusk (3.00 p.m. to 6.00 p.m.). Of the two peaks, the highest predation rate was observed at the dusk, suggesting that the copepods are remain mostly active at dusk.

### Predatory efficiencies of copepods on Aedes larvae

The predatory efficiencies also varied significantly among the copepods as suggested by the statistics of GLM (p<0.05). For both *Aedes* vectors, *M*. *leuckarti* showed the highest predatory efficiency of 17.45 and 16.75 for *Ae*. *aegypti* and *Ae*. *albopictus*, respectively, followed by *M*. *scrassus* and *C*. *vernalis*. As shown in Table 2, *C*. *languides* had the lowest predatory efficiency for *Ae*. *aegypti* (5.3) and (4.2) mosquitoes. As suggested by the Turkey’s pair-wise comparison, four significantly different subsets of copepods were identified based on the mean predation rates of *Ae*. *Aegypti*, as (a) *M*. *leuckarti*; (b) *M*. *scrassus*; (c) *C*. *vernalis and C*. *varicans* (d) *C*. *languides*. In case of *Ae*. *Albopictus*, the mean predation rates of all copepods were significantly different from each other (p<0.05 at the 5% level of significance). GLM also indicated that the effect of *Aedes* vectors on the predatory efficacy of copepods was non-significant (p>0.05) at the 5% level of significance.

**Table 2:** Mean predatory efficiencies of studied copepods on Aedes larvae (1st instar).

Copepod Species	Predatory Efficiency in terms of *Aedes* larval (1^st^ instar) consumption	
	*Ae*. *aegypti*	*Ae*. *albopictus*
*Mesocyclop leuckarti*	17.45±1.8 ^a^ (15.65–19.25)	16.75±1.6 ^a^ (15.15–18.35)
*Mesocyclop scrassus*	15.5±1.4 ^b^ (14.1–16.9)	14.2±1.7 ^b^ (12.5–15.9)
*Cyclops vernalis*	8.6±0.9 ^c^ (7.7–9.5)	9.8±0.8 ^c^ (9–10.6)
*Cyclops varicans*	7.6±0.7 ^c^ (6.9–8.3)	6.4±0.7 ^d^ (5.7–7.1)
*Cyclops languides*	5.3±0.6 ^d^ (4.7–5.9)	4.2±0.7 ^e^ (3.5–4.9)

**Note:** Values are Mean ± SE, range in parenthesis. Different superscript letters (from a to e) in a column show significant differences (p< 0.05) as suggested by General Linear Modelling followed by the Tukey’s pair wise comparison at 95% level of significance.

**Fig 4:**
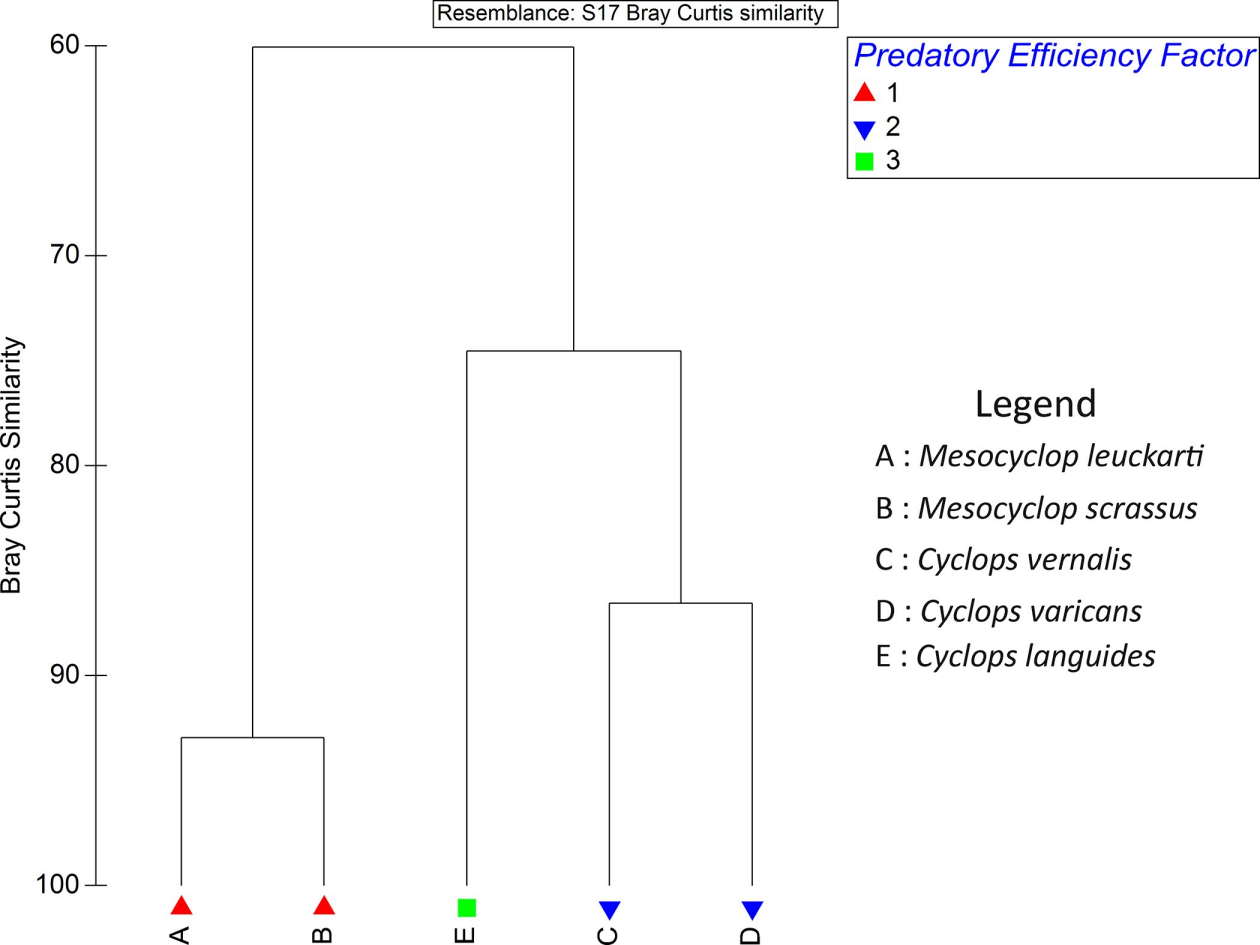
Dendrogram of the cluster analysis for the copepods based on predatory efficiency on *Aedes* larvae.

**Fig 5:**
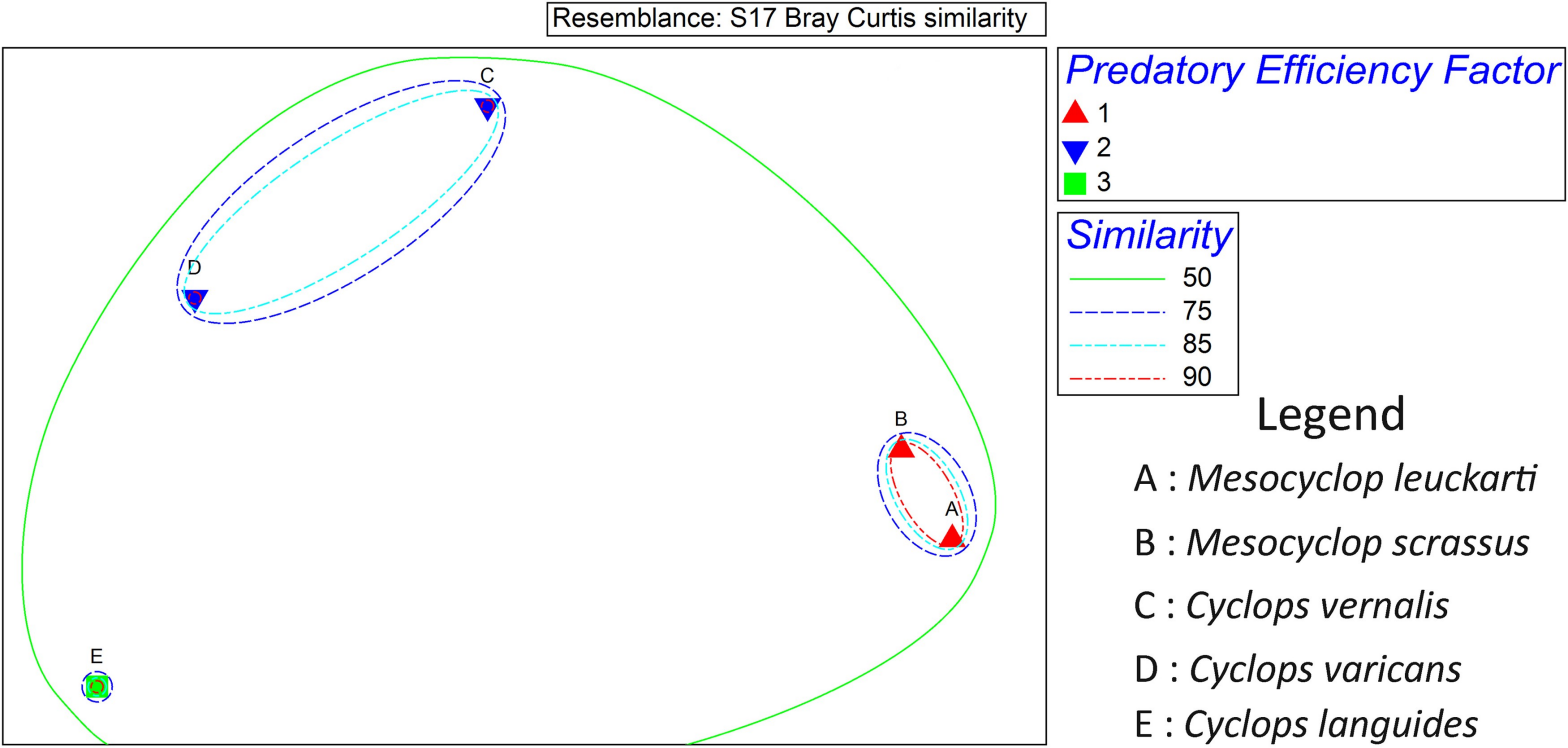
Multidimensional scaling (MDS) plot for the copepods based on predatory efficiency on *Aedes* larvae.

**Fig 6:**
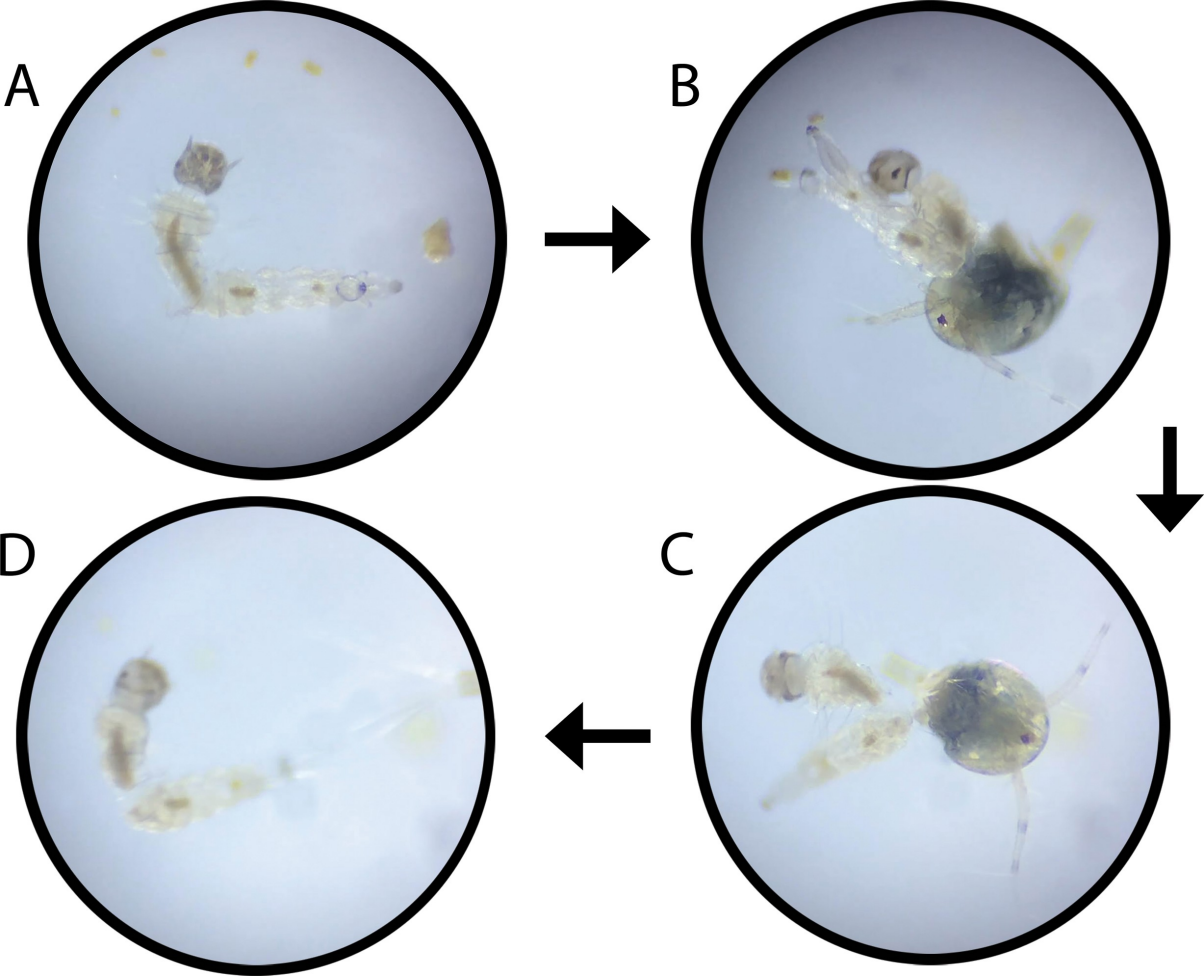
Predation of *Aedes* larvae by *Mesocyclop leuckarti*. (A) First instar *Aedes* larvae. (B) Capturing of *Aedes* larvae by *Mesocyclop leuckarti*. (C) Killing of *Aedes* larvae by *Mesocyclop leuckarti*. (D) Dead *Aedes* larvae.

Based on the overall predatory efficiency of copepods against *Ae*. *aegypti* and *Ae*. *albopictus*, three clusters of copepods were observed in the dendrogram of the cluster analysis at 74.5% similarity level (based on Bray Curtis Similarity). Both *M*. *leuckarti* and *M*. *scrassus*, with relatively high predatory efficacies were clustered together as the first cluster at a similarity level of 92.96%(based on Bray Curtis Similarity Resemblance), while *C*. *vernalis* and *C*. *varicans* composed the second cluster with 86.55% similarity. Meanwhile, *C*. *languides*, that denoted the lowest predation rates for both *Aedes* vectors, formed the third cluster (Fig 4). The distribution of the copepods into three clusters was further verified by the Analysis of Similarities (ANOSIM) at 5% level of significance with a Global R value of 0.96. On the other hand, Multi-Dimensional Scaling (MDS) plot also advocated formation of the above three clusters (Fig 5), confirming the results suggested by cluster analysis and ANOSIM. The predation of *Ae*. *aegypti* larvae by *M*. *leuckarti* is depicted in Fig 6.

## Discussion

Despite different approaches of vector control such as frequent use of larvicides, chemical treatment methods and mechanical source reduction, dengue still remains as the worst challenge faced by the health officials in Sri Lanka. Limitations in the above vector control methods have encouraged the VCE to search for alternative methods to be incorporated in to integrated vector management for dengue [38]. Among the available options, use of biological control agents such as copepods, to suppress *Aedes* vector populations is one of the sustainable and eco-conscious approaches.

All the tested copepod species, *C*. *languides*, *C*. *varicans*, *C*. *vernalis*, *M*. *leuckarti* and *M*. *scrassus* have been reported as predators of immature stages of mosquitoes including *Aedes*, *Anopheles* and *Culex* [25–27]. Among them *M*. *leuckarti* was the most efficient predator followed by *M*. *scrassus*. *M*. *leuckarti* is characterized by a high diversity of breeding habits including eutrophic lakes, ponds, paddy lands and small water pools consisting of high micro-algal productivity. On the other hand, *M*. *scrassus* is commonly found to inhabit rock pools near streams and rivers [39].

Being both a predator and competitor for mosquito larvae, *Mesocyclops* have a high potential to act as ideal biological controlling agents against numerous mosquito vectors including *Aedes* [40]. As highlighted by previous studies, a variety of copepods belonging to the *Mesocyclops* sp. such as, *M*. *thermocyclopoides* [41], *M*. *aspericornis* [42], *M*. *albidus* [43], *M*. *ongisetus* and *M*. *albidus* [27] have been reported as efficient biological control agents of mosquito larvae. The comparatively higher body size of the *Mesocyclops* copepods could be an additional factor that would have led to the higher predation rates than the other tested cyclopoids [40]. A study conducted by Bapna and Renapurkar [44], has investigated the predatory efficacy of *M*. *leuckarti* against first instar larvae of *Culex quinquefasciatus* and concluded that predatory potentiality increases with increasing prey density and hunger state of the predator, while it decreased with increase in water volume and choice of other zooplanktons. Meanwhile, a study conducted in Italy recommended *M*. *leuckarti* as an effective candidate for controlling *Ae*. *koreicus* and *Ae*. *albopictus* with more than 50% reductions of first instar larvae within 24 hours [45]. Therefore, maintaining a high prey density of *Aedes* larvae in a confined container (with a limited water volume) without other planktonic matter [46] will significantly enhance the predatory potential of *M*. *leuckarti*. Since, *Aedes* is a container breeder, the above requirements of high prey density in a limited volume of water will be naturally facilitated ensuring the practical feasibility of using *M*. *leuckarti* as a biological control agent.

Success of any biological control approach by using a natural predator is influenced by the ecological characteristics of the habitat [47–48]. In this case, the predators should be capable of inhabiting a wide range of habitats with different physico-chemical conditions. The diverse habitat range of *M*. *leuckarti* and *M*. *scrassus* would enable them to inhabit a variety of habitats making them effective predators of *Aedes* larvae. Even though, reports on the field application of *M*. *leuckarti* and *M*. *scrassus* against *Aedes* are lacking, *M*. *thermocyclopoides* of *Mesocyclops* genus has shown its ability to survive under three different climatic conditions for 2–6 months in different habitats such as bromeliad leaf axils and used tires, while effectively reducing the *Aedes* larval population upto 79, 90, and 99% in tropical dry, moderate and humid climates, respectively [47]. Further, *Mesocyclops* populations have shown the capability of being established in artificial containers such as cement tanks, drums, and big jars in Vietnam [48], which are also commonly found in Sri Lanka. *M*. *leuckarti* has been reported to survive in a wide range of water temperatures (ranging from 0–40°C) and pH values (pH 4.5–8) [49–50] enabling them to inhabit the available breeding habitats of *Aedes* with different physico-chemical properties. Therefore, it could be assumed that the local climatic conditions of Sri Lanka and the common breeding sites would fit ideally for the survival of *Mesocyclops* copepods under field conditions making them potential candidates for biological control of *Aedes* vectors.

Successful implementation of a copepod based dengue vector suppression programme requires self-sustenance of introduced copepods in artificial containers under urban and semi-urban environments. Several studies have highlighted the potential of *Mesocyclops* in establishing themselves in artificial containers as a routine colony [27, 48]. In addition, mass colonies of copepods are needed for the initial introduction of copepods into the breeding habitats of the target vector, *Aedes*. As emphasized by Surrez *et al*. [51], the reproduction of copepods is easy and inexpensive enabling them to be used by even countries with low economic conditions as effective controlling agents of dengue vectors. Further, copepods are known to survive for long periods of time even in artificial containers increasing the sustainability of the approach [27]. Therefore, use of copepods as biological control agents of immature stages of *Aedes*, bear less operational and capital costs, while it requires minimal labour for colony maintenance, highlighting their easy and cheap potential as mass-reared biological controlling agents [52]. Most successful application of copepods as biological control agents of dengue vectors has been conducted in Northern Vietnam targeting *Ae*. *aegypti* in 1993, which has resulted in zero dengue prevalence by 2000, in a large vicinity of surrounding areas [48]. Since then, copepods are used for biological control of *Aedes* by many communities including Vietnamese and Thailand [47].

However, application of copepods also has limitations since copepods are more successful predators of first and second instar mosquito larvae, while the older stages (third and fourth instars and pupae) may escape the predation by copepods, due to the swimming behavior and small size of the copepods [46, 53]. Therefore, the older immature stages of *Aedes*, present at the initial introduction of copepods may escape and release into the environment as adult mosquitoes. Application of minimum levels of larvicides may be a potential solution for this along with the introduction of copepods. A study conducted in Thailand by Kittayapong *et al*. [47], has utilized this principle in controlling dengue vectors as an integrated approach for vector management. However, for this to become a success the copepods of interest should be able to withstand the toxic effects caused by the chemical controlling agents being used. According to Marten *et al*. [27] and Marten [30] cyclopoid copepods have shown potential to predate upon the *Aedes* larve, even in the aquatic medium contaminated with larvicides and adulticides such as permethrin and methoprene. Therefore, an integrated approach where a compatible larvicide is applied along with the initial introduction of cyclopoids, would enhance the suppression of *Aedes* populations, since the chemical agent can eradicate the existing mosquito larvae immediately, while the cyclopoids predate on the new larvae that appear.

Based on the findings, cyclopoids, especially *M*. *leuckarti* and *M*. *scrassus* shows promising results to be used as potential candidates for biological control of *Aedes* vectors in Sri Lanka. The successful application of copepods by many other counties with similar environmental and socio-economic settings further enhances the applicability of the approach as a low-cost, environmental friendly and sustainable method for vector control [47, 52]. Therefore, it is suggested to conduct further research on the bio control efficacy of *M*. *leuckarti* and *M*. *scrassus* under semi-field and field conditions to evaluate the practical feasibility of suppressing *Aedes* vector populations by the use of copepods within Sri Lanka. Further, copepods may be used as a key controlling method in Integrated Vector Management (IVM) approaches to control the incidence of dengue outbreaks.

### Conclusions

Predation rates of the five studied copepods varied significantly along with the predatory efficacies, while *M*. *leuckarti* had the highest predation of *Ae*. *aegypti* (34.9±1.80) and *Ae*. *albopictus* (33.5±1.06) within 24 hours along with predatory efficiencies of 17.45 and 16.75 for *Ae*. *aegypti* and *Ae*. *albopictus*, respectively. Even though the impact of *Aedes* larval species on the predation rates was significant (p<0.05), the predation efficiencies of copepods were not influenced by the species of the vectors (p>0.05). In case of temporal dynamics in the predation rates, a relatively high predation was observed at dusk on both *Aedes* vectors by the studied copepods. Based on these findings, both *M*. *leuckarti* and *M*. *scrassus* with relatively high predation efficacies on *Ae*. *aegypti* and *Ae*. *albopictus* could be recommended as ideal candidates for biological control as a more ecofriendly, low cost and sustainable method for management of dengue epidemics within Sri Lanka. Further studies on the predatory efficacy of above copepods could be suggested under semi field and field settings.

## Supporting information

S1 File Dataset used to develop Fig 2 and Fig 3. (PDF)

## Abbreviations

ANOSIM: Analysis of Similarities
DF: Dengue Fever
DHF: Dengue Haemorrhagic Fever
GLM: General Linear Model
IVM: Integrated Vector Management
MDS: Multi-Dimensional Scaling
PRIMER: Plymouth Routines in Multivariate Ecological Research
SIT: Sterile Insect Technique
SPSS: Statistical Package for the Social Sciences
WHO: World Health Organization
