# Supplementary material for: Predatory efficacy of five locally available copepods on Aedes larvae under laboratory settings: An approach towards bio-control of dengue in Sri Lanka
Source: PLoS One. 2019 May 28;14(5):e0216140. doi: 10.1371/journal.pone.0216140 (PMC6538144; doi:10.1371/journal.pone.0216140)
Supplement: S1 File — (PDF) [file pone.0216140.s001.pdf]

S1 File. Dataset used to develop Fig 2 and Fig. 3

S1 File Table 1. Temporal variation of the predation rates of studied copepods on *Ae. aegypti* larvae

**Copepod Species**	**9.00-12.00 am**	**12.00-3.00 pm**	**3.00-6.00 pm**	**6.00-9.00 pm**	**9.00-12.00 pm**	**12.00-3.00 am**	**3.00-6.00 am**	**6.00-9.00 am**
Mesocyclop scrassus	3.4	2.8	4.2	6.3	4.3	2	2.1	5.9
Cyclops varicans	1.5	1.4	2	2.9	2.2	1.4	1	2.8
Cyclops languides	1.4	0.8	1.2	2.8	1.4	1.3	0.3	1.4
Mesocyclop leuckarti	4.1	3.2	4.9	7.5	3.1	2.3	2.7	7.1
Cyclops vernalis	2.7	1.2	2	4.2	2.2	1.7	0.8	2.3

S1 File Table 2. Temporal variation of the predation rates of studied copepods on *Ae. albopictus* larvae

**Copepod Species**	**9.00-12.00 am**	**12.00-3.00 pm**	**3.00-6.00 pm**	**6.00-9.00 pm**	**9.00-12.00 pm**	**12.00-3.00 am**	**3.00-6.00 am**	**6.00-9.00 am**
Mesocyclop scrassus	2.7	2.4	3.8	5.3	4.8	1.4	2.7	5.3
Cyclops varicans	0.4	1.4	1.8	2.9	1.3	0.6	1.2	3.2
Cyclops languides	0.9	0.6	0.6	2.1	0.8	0.9	0.4	2.1
Mesocyclop leuckarti	3.2	3.5	3.9	6.3	3.8	2.5	4.1	6.2
Cyclops vernalis	0.6	1.3	2.1	3.9	2.4	2.7	2.1	4.5
